# Nutritional Correlates of Perceived Stress among University Students in Egypt

**DOI:** 10.3390/ijerph121114164

**Published:** 2015-11-06

**Authors:** Walid El Ansari, Gabriele Berg-Beckhoff

**Affiliations:** 1 Faculty of Sport, Health and Social Care, University of Gloucestershire, Gloucester GL2 9HW, UK; 2 Unit for Health Promotion Research, Institute of Public Health, University of Southern Denmark, Niels Bohrs Vej 9-10, 6700 Esbjerg, Denmark; E-Mail: gberg-beckhoff@health.sdu.dk

**Keywords:** stress and food intake, student health, healthy eating, dietary guidelines adherence

## Abstract

Food intake choice and amount might change with stress. However, this has not been examined among Egyptian students. We examined students’ stress levels, its correlation with their consumption of a range of food groups, and adherence to dietary guidelines. A cross sectional survey (*N* = 2810 undergraduates at 11 faculties at Assiut University, Egypt) assessed two composite food intake pattern scores (one unhealthy: sweets, cakes, snacks; and a healthy one: fruits and vegetables), and two indicators of healthy eating (subjective importance of healthy eating; and dietary guideline adherence index). Multiple linear regression tested the associations of stress with two food intake pattern scores and two indicators of healthy eating, controlling for six potential confounders for the sample and separately for males and females. Higher perceived stress score was significantly associated with less frequent food intake of fruit and vegetables in males and females. The association was more pronounced among males than in females. No significant association was observed between the sweets cakes and snacks score and stress. Of the two indicators of healthy eating, the dietary guideline adherence index was not associated with stress, while the subjective judgment of healthy eating was consistently negatively associated with stress. Stress related decreased-eating was present. Recent studies suggest that stress could be associated with either decreased or increased eating depending on the study population, food group, and type of stressor. Further research is necessary to understand stress related over- and undereating.

## 1. Introduction

Entry to college, whilst exciting, can be stressful for some students. This is due to being away from their family home (sometimes for the first time), new socializations, personal expectations, peer competition, the need to achieve good grades, trying to achieve academic success despite financial constraints, and distress about failing and repeating their course [1]. College students’ stress levels are important as students use different strategies to cope with stress (e.g., alcohol, smoking, illicit drug/s use) that might include unhealthy eating. For many people, eating in response to negative emotions or stress is quite common, and may be considered as an emotional relief and a form of maladaptive coping [2]. Studies of stress and food choice found that individuals experiencing periods of stress overate food items they would customarily avoid [3]. However, findings of the association between stress and food selection seem partly conflicting [4,5,6,7]. For a minority of individuals stress or reactions to stress decreases food intake and is associated with weight loss, however, in most stressed people, food intake, particular energy dense, sweet or fat foods, is increased [8].

To date, there seems to be no clear understanding of stress-related under- and overeating. Cultural or religious behavior might be a potential explanation. In Western countries, it is documented that emotional symptoms and stress affect eating behavior and food intake. However, very little studies have been undertaken on the relationships between dietary habits, dietary guidelines adherence and stress in the Eastern Mediterranean Region. Although patchy research on food habits has been conducted in Saudi Arabia [9,10] and on the impact of life style on the nutritional status of medical students in Egypt [11], to the best of our knowledge, no study has explicitly considered the association between food habits and stress. Researchers have also voiced that little is known about the association of stress with the frequency of consumption of various food groups among college/ university students [12,13].

Therefore the current study aimed to correlate specific nutritional behaviour with perceived stress. The research contributes to the existing knowledge by examining the association between perceived stress of male and female university students in Egypt and their self-reported food intake, subjective importance of healthy eating, and dietary guidelines adherence.

## 2. Methods

### 2.1. Study Design, Sample and Data Collection

This cross sectional study comprised self-administered questionnaires that were distributed to students attending lectures of randomly selected courses at the University of Assiut, Egypt. The University Research Committee approved the study ethics, and data were collected at the same time (2009–2010, between the months of September and June) from all participating faculties. Participation was voluntary, anonymous and data were confidential. Data comprised 3271 students, out of which 461 participants were excluded due to missing data of the important variables (perceived stress, food frequency questionnaire, and gender), leaving 1483 females (52.8%) and 1327 male (47.2%) from 11 faculties (Business, Engineering, Education, Arts, Social Work, Sciences, Physical Education, Computers and Information, Veterinary Medicine, Specific Education, and Agriculture). The response rate was quite high (about 90%), probably due to the general culture of university students in Egypt, the fact that the survey was administered usually in the last 10 min of lectures that students attended, thus facilitating students’ participation, and that it was a paper and pencil questionnaire (*i.e.*, not an online survey which could yield lower response rates).

The study was a general student health and wellbeing survey similar to studies implemented in several countries [14]. It included self-reported socio-demographic information, nutritional habits (consumption frequency of 12 food groups), two food intake pattern scores, two indicators of healthy eating (*subjective* importance of healthy eating; and *objective* computed healthy eating guideline adherence index); perceived stress; and further covariates.

#### 2.1.1. Perceived Stress Scale

Cohen’s Perceived Stress Scale (PSS) in its four-item short form [15] assessed the degree to which situations in one’s life over the past month are appraised as stressful. (5-point scale: 0 = “never”, 1 = “almost never”, 2 = “sometimes”, 3 = “fairly often”, 4 = “very often”). The responses were summed up so that higher scores indicated more perceived stress. Cronbach’s Alpha was 0.47. The same questionnaire used for Finnish University students revealed a Cronbach’s Alpha 0.76 [16].

#### 2.1.2. Assessment of Food Intake

Students self-reported their food intake habits in a food frequency questionnaire (FFQ) that measured their usual consumption of 12 food groups (each food group individually) (5 = “several times a day”, 4 = “daily”, 3 = “several times a week”, 2 = “1–4 times a month”, and 1 = “never”) (Table 1). The instrument was based on pre-existing food frequency questionnaires, adapted for the study [17] and analogous to other validated FFQs [18,19]. The categories “several times a week” and “daily” were collapsed together. Then, in order to bring together (bundle) each of the healthy and less healthy food groups, we calculated two composite food intake pattern scores. The first score was for the less healthy options—sweets, cake/cookies, and snacks where their relevant scales were added; and the second food intake pattern score was for the healthier options—fruits, and raw and cooked vegetables where their relevant scales were added.

#### 2.1.3. Dietary Guideline Adherence Index

Using the students’ responses to the FFQ, a dietary guideline adherence index with maximum of eight points (eight guidelines) was computed, derived from eight foods: (1) sweets, cookies and snacks; (2) fast food/ canned food; (3) lemonade/soft drinks; (4) fruits; (5) salad and raw vegetables; (6) cooked vegetables; (7) meat; and; (8) fish. For sweets, cake/cookies, snacks, fast food/canned food and lemonade/soft drinks, no specific guidelines exist; hence we employed “1–4 times a month” and “never” as recommended. To consider all sweets, cake/cookies and snacks together, we used the above composite food intake pattern score for “sweets, cookies and snacks score”, and healthy eating was considered present if this score was ≤ 6 corresponding to intake of these items “less often than 1–4 times a month” three times. Each of the fast food/canned food and lemonade/soft drinks were included as individual items in computing the objective guideline adherence index. For the remaining food groups, we used the WHO dietary guidelines recommendations for Eastern Mediterranean region [20]. Consequently, for the number of daily fruit, raw and cooked vegetables servings, the cutoff was “daily” or “several times a day”. For meat, the cutoff was “less than daily”; and for fish “several times/week” was employed as a cutoff. Milk and cereals were not included in computing the healthy eating adherence index. Milk consumption is healthy if lactose intolerance is not present, a condition that appears quite often (74%) among Egyptian children [21]; and the information about cereals is generally too unspecific in order to categorize as healthy or unhealthy nutrition.

#### 2.1.4. Importance of Eating Healthy

“How important is for you to eat healthy?” (1 = “Not at all important”, 5 = “very important”).

#### 2.1.5. Potential Confounders That Were Controlled for in the Regression Analysis

These included: (1) age; (2) gender; (3) respondent’s subjective economic situation (“How sufficient is your income?” coded into sufficient *vs.* not sufficient); (4) living situation/arrangements during university terms (“Where do you live during university/college term time?” coded into living at parental home *vs.* not living at home); (5) Vigorous and moderate physical activity. Vigorous physical activity was measured using the question: “On how many of the past 7 days did you participate in vigorous exercise for at least 20 min?” Participants answered with 0–7 days. We used a cut-off of ≥3 days/week as adherence to the physical activity guidelines [22]. Moderate physical activity was measured using the question: (“On how many of the past 7 days did you participate in moderate exercise for at least 30 min?” Participants answered with 0–7 days. We used a cut-off of ≥5 days/week as adherence to the physical activity guidelines [22]; (6) Faculties (business, engineering and computer science, education and physical education, arts, social science, science, veterinary medicine, and other); and, (7) body mass index (BMI), calculated from measured weight and height (kg/m^2^).

### 2.2. Statistical Analysis

Analysis was conducted in SAS Version 9.4 (SAS Institute Inc., Cary, NC, USA, *p* set at < 0.05). For descriptions, spearman rank coefficient quantified the correlation between each of the nutrition related variables and the perceived stress score. Multiple linear regression models analysed the effects of the different nutrition related exposures on perceived stress. Data were adjusted for age, sex, living situation (accommodation during term time), economic situation, vigorous and moderate physical activity, Faculty, and BMI. Model assumptions were graphically tested and fulfilled for all models. We tested for interaction between sex and food pattern on perceived stress. We found significant interaction between both food intake pattern score (fruit, and raw and cooked vegetable and sweets, cookies and snacks) and gender, but no interaction was found between subjective importance of healthy eating and dietary guideline adherence index and gender. For better interpretation, we present all results stratified by gender.

## 3. Results

Table 1 shows the socio demographic and lifestyle characteristics, as well as intake of 12 food groups for the whole sample and by gender.

For both genders, the age ranged from 16 to 30 years. More than half of the students did not live with their parents; and this was more common in females than in males. Around 75% of the sample felt they had sufficient money. Twenty five percent of men and 32% of women were overweight (BMI > 25 kg/m^2^). Whilst females ate sweets and cookies more often than males, both genders ate more or less similar amounts of fruits. Males ate more salad and raw vegetables, while females consumed more cooked vegetables. Daily fish consumption was uncommon and similarly distributed among both genders; 3% of women and 3.4% of men consumed fish daily or several times a day.

**Table 1:** Socio demographic and lifestyle characteristics and intake of 12 food groups by gender.

Variable	Females		Males	
	*N*	%	*N*	%
	1483	52.8	1327	47.2
**Age group** (years)				
16–18	756	51.0	469	35.3
19–21	695	46.9	751	56.6
22–30	20	1.4	89	6.7
Missing	12	0.8	18	1.4
**Living situation** (accommodation)				
Living with parents	482	32.5	572	43.1
Not living with parents	982	66.2	730	55.0
Missing	19	1.3	25	1.9
**Economic situation** (income)				
Always/Mostly sufficient	1176	79.3	956	72.1
Always/Mostly insufficient	268	18.1	342	25.8
Missing	39	2.6	29	2.1
**Physical activity (PA)**				
Vigorous PA (Always/Mostly sufficient)	218	14.7	273	20.6
Only moderate PA (Always/Mostly sufficient)	329	22.2	270	20.4
Any of both PA (Always/Mostly insufficient)	730	49.2	631	47.6
Missing	206	13.9	153	11.5
**BMI**					
Below normal (<20 kg/m^2^)	198	13.6	237	18.2
Normal (20–25 kg/m^2^)	778	53.7	737	56.6
Overweight (>25 kg/m^2^)	474	32.7	328	25.2
**Faculty**				
Business	153	10.3	365	27.5
Engineer and computer science	221	14.9	386	29.1
Education and physical education	333	22.5	214	16.1
Arts	289	10.3	100	7.5
Social science	178	12.0	96	7.2
Science	110	7.4	83	6.2
Veterinary medicine	82	5.5	31	2.3
Other	117	7.9	52	3.9
**Food frequency questionnaire** (FFQ) *				
Sweets	320	21.6	154	11.6
Cake, cookies	286	19.3	189	14.2
Snacks	588	39.7	306	23.6
Fresh fruits	323	21.8	273	20.6
Salad, raw vegetables	261	17.6	372	28.0
Cooked vegetables	630	42.5	428	32.3
Fast food, canned food	293	19.7	410	30.9
Lemonade, soft drinks	272	18.3	278	21.0
Meat, sausages	238	16.5	202	15.2
Fish, sea food	44	3.0	45	3.4
Milk and milk products	252	17.0	288	21.7
Cereals and their products	1053	71.0	962	72.5

FFQ = food frequency questionnaire; * percentages calculated for intake of “several times per day” or “daily”.

Table 2 depicts the perceived stress levels, stressors, two food intake pattern scores, subjective importance of healthy eating, and objective computed healthy eating adherence index of Egyptian university students by gender. Women were, as expected, more stressed than men. Only marginal gender differences were observed with regard to fruit and vegetable consumption, but females ate more sweets, cookies and snacks than men. No gender difference was present with regard to healthy eating habits. Nearly all participants agreed that healthy eating was important, where the mean score for both genders was > 4 (out of a maximum score of 5). As for the healthy eating adherence index, men and women adhered to a mean 3 out of 8 considered guidelines (foods).

**Table 2:** Stress and eating habits of Egyptian university students by gender.

Variable	Females		Males		*p* Value
	*N*	Mean (SD)	*N*	Mean (SD)	
Perceived stress *	1483	9.14 (3.06)	1327	8.04 (2.85)	<0.001
Food intake pattern score					
Fruit, and raw & cooked vegetable ^‡^	1483	8.63 (2.26)	1327	8.82 (2.19)	0.01
Sweets, cookies and snacks ^‡^	1483	8.79 (2.48)	1327	7.81 (2.27)	<0.001
Healthy eating					
Subjective importance of healthy eating ^§^	1434	4.33 (1.05)	1274	4.36 (0.99)	0.77
Dietary guideline adherence index ^||^	1483	3.05 (1.22)	1327	3.02 (1.26)	0.34

*p* values based on Wilcoxon ranks sum test; SD = standard deviation; * range 0–16, higher values correspond to more perceived stress; ^‡^ range: 3–15 each, scores increase as more is reported to be eaten; ^§^ range: 1–5, higher values indicate higher importance of healthy eating; ^||^ range: 1–8, each point increase presents an additional food group that exhibited adherence to dietary guidelines.

Table 3 shows the correlation coefficients between stress and all nutritional variables (FFQ, two food intake pattern sores, subjective importance of healthy eating, and dietary guideline adherence index) stratified for females and for males. All the FFQ food groups were negatively associated with stress, showing that generally, perceived stress was associated with less food intake. These results were also confirmed by the two food intake pattern scores, but it was more pronounced and more stringent with the fruit, raw and cooked vegetables score than with the sweet, cookies and snacks score. The subjective importance of healthy eating was also negatively associated with increased perceived stress. There was no correlation between the computed healthy eating adherence index and perceived stress.

**Table 3:** Correlation between stress and food frequency items derived from FFQ, food intake pattern sores, and healthy eating indices.

Variable	Perceived Stress			
	Females		Males	
	Correlation	*p* Value	Correlation	*p* Value
**FFQ**				
Sweets	−0.00	0.89	**−0.09**	**<0.01**
Cake/cookies	−0.01	0.78	**−0.07**	**0.01**
Snacks	−0.04	0.14	−0.04	0.09
Fresh fruits	**−0.06**	**0.02**	**−0.16**	**<0.01**
Salad, raw vegetables	**−0.07**	**0.01**	**−0.15**	**<0.01**
Cooked vegetables	−0.03	0.21	**−0.07**	**0.01**
Fast food, canned food	−0.02	0.34	−0.00	0.95
Lemonade, soft drinks	**−0.07**	**0.01**	**−0.11**	**<0.01**
Meat, sausages	**−0.07**	**<0.01**	**−0.10**	**<0.01**
Fish, sea food	−0.03	0.24	−0.04	0.08
Milk and milk products	**−0.07**	**0.01**	**−0.11**	**<0.01**
Cereals and their products	0.02	0.32	**−0.07**	**0.01**
**Food intake pattern scores**				
Sweets, cookies & snacks	0.01	0.78	**−0.09**	**<0.01**
Fruits, and raw & cooked vegetables	**−0.08**	**<0.01**	**−0.17**	**<0.01**
**Healthy eating**				
Subjective importance of healthy eating	**−0.11**	**<0.01**	**−0.10**	**<0.01**
Dietary guideline adherence index	0.00	0.98	−0.02	0.54

Spearman correlation coefficient; bolded cells indicate statistical significance.

Table 4 (multiple linear regression models) depicts the nutritional correlates of perceived stress for the whole sample and by gender. Overall, higher intake of sweets, cookies and snacks was not significantly associated with perceived stress. Higher intake of fruits and vegetables was associated with a lower perceived stress score. This latter association was observed with regard to the overall sample and male students, but it was less pronounced for females. Increased subjective importance of healthy eating was significantly associated with a decrease in perceived stress for the whole sample, and also separately for females and for males. There were no association between perceived stress and the dietary guideline adherence index.

**Table 4:** Multiple linear regression model on nutritional correlates of perceived stress: two food pattern scores and two healthy eating indices.

Variable	Perceived Stress		
	Whole Sample	Females	Males
	β (95% CI)	β (95% CI)	β (95% CI)
Food intake pattern score			
Sweets, cookies & snacks	−0.02 (−0.07; 0.03)	0.01 (−0.06; 0.07)	−0.06 (−0.12; 0.01)
Fruits, and raw & cooked vegetables	**−0.12 (−0.17; −0.07)**	−0.06 (−0.13; 0.02)	**−0.19 (−0.26;−0.11)**
Healthy eating			
Subjective importance of healthy eating	**−0.30 (−0.41; −0.19)**	**−0.30 (−0.45; −0.14)**	**−0.30 (−0.46; −0.14)**
Objective healthy eating adherence index	−0.02 (−0.11; 0.07)	0.00 (−0.13; 0.13)	−0.04 (−0.17; 0.08)

All models adjusted for age, living situation, economic situation, vigorous and moderate physical activity, faculty, and BMI, and the model for the whole sample is additionally adjusted for sex; bolded cells indicate statistical significance (*p* < 0.05).

## 4. Discussion

Higher food intake was associated with lower perceived stress across this sample of Egyptian undergraduates. This association was true for all the FFQ foods groups, even though the association was not significant for snacks, fish and fast food. However, when controlled for the many potential confounders e.g., age, living situation, economic situation, physical activity, and BMI, this negative association disappeared for sweets, cakes and snacks but remained significant for fruit and vegetables. Upon the first inspection, our observed finding contrasts with previous research [3,4,5,11,12,23,24,25]; but a recent review found that stress results in either increased or reduced food intake depending on the types of external or psychological stressors [26]. Similarly, another review of emotions and eating habits summarized the findings of eight surveys in terms of changes of eating habits in response to emotional stress. All the reviewed surveys reported stress related over- and under- eating, with a generally lower percentage of more appetite due to stress (4%–55%), and a higher percentage of having less appetite due to stress (42%–70%) [27]. Particularly in terms of consuming fruits and vegetables, our findings of eating behavior and stress support that higher stress is associated with lower consumption of fruits and vegetables [6,23,28]. In addition, an experimental study of the effects of academic examination stress on eating behaviors among students in the UK found no effects of exam stress on food intake [7]. Hence, our findings agree with and are confirmed by some of the more recent studies. It is worth exploring the potential underlying mechanisms for our observed negative association between stress and food intake. It has been suggested that stress eating habits depend on the types of stressors [24]. For instance, exam related stress at universities might need to be interpreted differently than overall lifetime or chronic stress situations.

In terms of eating habits, research has observed that eating habits and stress might be associated in different ways depending on the type of eating habits [26]. There are, for example, different associations between stress and food intake that are seen among emotional eaters, restraint eaters and uncontrolled eaters [24,27,29]. Given that the effect of stress among these diverse eating behavior subgroups is not homogenous, and in fact differed in the cited publications, such heterogeneity in the stress-food intake association suggested at minimum that there were sub populations or “vulnerable groups”, where the association between food habits and stress was “not traditional”. In addition, other potential features that could contribute to explaining the different associations between stress and food habits might relate to the overall stress type/load [29], or alternatively to cultural and sociological background, or religion. Such aspects seem to be very under-researched in the literature. Future research would benefit from exploring the underlying mechanism/s for different (positive or negative) stress-food intake relationships, and examining the potential predictors that might assist in the differentiation between “stress over-eaters” and “stress under-eaters”.

In addition, we examined the association between perceived stress and the extent of adherence to healthy eating guidelines. Our computed healthy eating guideline adherence index was not at all associated with perceived stress in this sample of Egypt undergraduates. To date, only very few studies have examined the association between adherence to nutritional guidelines and stress. A cross sectional study among a community-based sample of African Americans showed that more perceived stress was associated with haphazard planning of meals, to conclude that during stressful periods, individuals were less likely to plan their meals carefully [24]. Whilst over-eating sweets and snacks, meat and fatty products is unhealthy; over-eating fruit and vegetables is healthy. All things considered, it is the combination of all these different food intake patterns that ultimately defines unhealthy or healthy eating habits. In our data, all the FFQ foods were under-eaten by students who perceived stress. A cross sectional survey among university students in the UK [28] found both overeating of snacks, sweets and fast food but also under eating of fruit and vegetables by those reporting high stress levels, which might suggest that nutritional guideline adherence index would be worse among persons with higher perceived stress. Further research would be beneficial to clarify the relationships between food guideline adherence and stress.

In terms of the subjective judgment/importance of healthy eating, we observed that this was most pronounced, consistent across males and females, and negatively associated with perceived stress: the higher the stress level—the less important healthy eating was judged. Our finding is in line with Sim’s interpretation that during stressful periods, individuals were less likely to plan their meals carefully [24]. However, an interesting point to note is the miscongruency between the subjective importance of healthy eating (most significantly associated with perceived stress) on the one hand; and the objective computed healthy eating guideline adherence (no association with perceived stress at all) on the other hand.

As for gender, we observed higher stress levels amongst females (Table 2), in support of research in Egypt that reported that female gender was associated with stress [30]. Despite this finding, we found that the correlations between stress and food frequency items (derived from FFQ), food intake pattern sores, and healthy eating indices were generally lower among females than males (Table 3) (association was more pronounced among male students). Likewise, in the regression analysis, higher intake of fruits and vegetables was associated with a lower perceived stress, but this was less pronounced among female students. Our findings support research on stress and dietary behaviour among first-year university students in Australia that found a distinct difference in food selection patterns between stressed male and female students, with stress being a more significant predictor of unhealthy food selection among male students [31]. Future research is needed, perhaps employing qualitative methodologies to uncover how stress and eating behavior are associated among university populations of young adults.

This study has limitations, and generalization of the findings needs to be cautious. Self-reporting of perceived stress and dietary food consumption might be subject to sociability and social desirability. As a cross-sectional survey, the direction of the association between food consumption and perceived stress cannot be ascertained. The semi quantitative FFQ should be interpreted with caution, and as we did not assess serving sizes, it does not allow the calculation of the precise amounts of food intakes. Further, there is no information on the validity of this specific FFQ among Egyptian students. Nonetheless, the tool was comparable to other published food frequency questionnaires that have been validated [18,19]. However, analyses of the food pattern scores and the dietary adherence index were based on the food intake frequencies (*i.e.*, how often students reported that they ate a given food). Due to the cross sectional study design, and the coarse assessment of the exposure, future research is necessary to deepen the understanding of these issues, and should focus on follow up/longitudinal study designs that employ validated and sophisticated assessments of the dietary habits. Our response rate was below 100%, and it could have been that students with higher stress levels might not have been at the university on the data collection days and might have had no further opportunity to participate in the study. It would have been useful to have had any available information on our non-responders in order to compare them with those who responded in an attempt to detect any differences in their characteristics or bias in the responses of the sample. Despite these limitations, the study’s main strengths include the use of a large sample, with high response rate, across many faculties, and comprising a broad variety of disciplines. Very little research has been undertaken in the Eastern Mediterranean Region on the dietary habits, dietary guidelines adherence and their relationships with stress. Less is known about the association of stress with the frequency of consumption of various food groups among college/ university students. We are not aware of previous research in Egypt that has assessed such relationships in detail.

## 5. Conclusions

In the current sample, women were more stressed than men. Only marginal gender differences were apparent with regard to fruit and vegetable consumption, but females ate more sweets, cookies and snacks than men. No gender difference was present with regard to healthy eating habits. Higher fruits and vegetables intake was associated with a lower perceived stress score. This latter association was seen with regard to the overall sample and among males, but it was less pronounced for females.

Stress-related decreased eating was present across this Egyptian sample. Recent studies seem to suggest that stress could be associated with both, eating less and/or eating more depending on several factors e.g., study population, food group, and type of stressor. Further research is necessary to differentiate between stress related over- and under-eating.

The lower subjective judgment of healthy eating while people are perceiving stress implies that during stressful periods, individuals might be less likely to plan their meals carefully. However, this does not seem to play a role in terms of their adherence to nutritional guidelines because adherence was not associated with perceived stress. Further research could outline the potential predictors in order to differentiate stress related under and over-eater, and to deepen our understanding of the association between stress related eating habits and nutritional recommendations across student populations in different faculties and countries, and by the type of stressor/s and other characteristics of the diet patterns.

## References

[1] Chan G.C., Koh D. (2007). Understanding the psychosocial and physical work environment in a Singapore medical school. Singapore Med. J..

[2] Manzoni G.M., Pagnini F., Gorini A., Preziosa A., Castelnuovo G., Molinari E., Riva G. (2009). Can relaxation training reduce emotional eating in women with obesity? An exploratory study with 3 months of follow-up. J. Am. Diet. Assoc..

[3] Zellner D.A., Saito S., Gonzalez J. (2007). The effect of stress on men’s food selection. Appetite.

[4] McCann B.S., Warnick G.R., Knopp R.H. (1990). Changes in plasma lipids and dietary intake accompanying shifts in perceived work-load and stress. Psychosom. Med..

[5] Michaud C., Kahn J.P., Musse N., Burlet C., Nicolas J.P., MeJean L. (1990). Relationships between a critical life event and eating behavior in highschool students. Stress Med..

[6] Oliver G., Wardle J. (1998). Perceived effects of stress on food choice. Physiol. Behav..

[7] Pollard T.M., Steptoe A., Canaan L., Davies G.J., Wardle J. (1995). Effects of academic examination stress on eating behavior and blood lipid levels. Int. J. Behav. Med..

[8] Epel E., Jimenez S., Brownell K., Stroud L., Stoney C., Niaura R. (2004). Are stress eaters at risk for the metabolic syndrome?. Ann. N. Y. Acad. Sci..

[9] El Hamid Hussein R.A. (2014). Socioeconomic status and dietary habits as predictors of home breakfast skipping in young women. J. Egypt Public Health Assoc..

[10] AL-Qauhiz N.M. (2010). Obesity among Saudi Female University Students: Dietary Habits and Health Behaviors. J. Egypt Public Health Assoc..

[11] Bakr E.M., Ismail N.A., Mahaba H.M. (2002). Impact of life style on the nutritional status of medical students at Ain Shams University. J. Egypt Public Health Assoc..

[12] Mikolajczyk R.T., El Ansari W., Maxwell A.E. (2009). Food consumption frequency and perceived stress and depressive symptoms among students in three European countries. Nutr. J..

[13] Liu C., Xie B., Chou C.P., Koprowski C., Zhou D., Palmer P., Sun P., Guo Q., Duan L., Sun X., Anderson Johnson C. (2007). Perceived stress, depression and food consumption frequency in the college students of China Seven Cities. Physiol. Behav..

[14] El Ansari W., Dibba E., Stock C. (2014). Body image concerns: Levels, correlates and gender differences among students in the United Kingdom. Cent. Eur. J. Public Health.

[15] Cohen S., Kamarck T., Mermelstein R. (1983). A global measure of perceived stress. J. Health Soc. Behav..

[16] El Ansari W., Suominen S., Berg-Beckhoff G. (2015). Mood and food at the university of Turku in Finland: Nutritional correlates of perceived stress are most pronounced among overweight students. Int. J. Public Health.

[17] El Ansari W., Stock C., Mikolajczyk R.T. (2012). Relationships between food consumption and living arrangements among university students in four European countries—A cross-sectional study. Nutr. J..

[18] Osler M., Heitmann B.L. (1996). The validity of a short food frequency questionnaire and its ability to measure changes in food intake: A longitudinal study. Int. J. Epidemiol..

[19] Roddam A.W., Spencer E., Banks E., Beral V., Reeves G., Appleby P., Barnes I., Whiteman D.C., Key T.J. (2005). Reproducibility of a short semi-quantitative food group questionnaire and its performance in estimating nutrient intake compared with a 7-day diet diary in the Million Women Study. Public Health Nutr..

[20] WHO (2012). Promoting a Healthy Diet for the WHO Eastern Mediterranean Region. Cairo: WHO, Regional Office for Eastern Mediterranean.

[21] Abuhamdah S.M.A., Oriquat G.A., Saleem T.H., Hassan M.H. (2013). Prevalence of Lactose Intolerance in Primary School Children in Qena Governorate, Egypt. Jordan J. Biol. Sci..

[22] Haskell W.L., Lee I.M., Pate R.R., Powell K.E., Blair S.N., Franklin B.A., Macera C.A., Heath G.W., Thompson P.D., Bauman A. (2007). A physical activity and public health: Updated recommendation for adults from the American College of Sports Medicine and the American Heart Association. Circulation.

[23] Groesz L.M., McCoy S., Carl J., Saslow L., Stewart J., Adler N., Laraia B., Epel E. (2012). What is eating you? Stress and the drive to eat. Appetite.

[24] Sims R., Gordon S., Garcia W., Clark E., Monye D., Callender C., Campbell A. (2008). Perceived stress and eating behaviors in a community-based sample of African Americans. Eat. Behav..

[25] Lääskeläinen A., Nevanperä N., Remes J., Fahkonen F., Järvelin M.R., Laitinen J. (2014). Stress related eating, obesity and associated behavioural traits in adolescents: A prospective population based cohort study. BMC Public Health.

[26] Singh M. (2014). Mood, food and obesity. Front. Psychol..

[27] Macht M. (2008). How emotions affect eating: A five-way model. Appetite.

[28] El Ansari W., Adetunji H., Oskrochi R. (2014). Food and mental health: Relationship between food and perceived stress and depressive symptoms among university students in the United Kingdom. Cent. Eur. J. Public Health.

[29] Wallis D.J., Hetherington M.M. (2008). Emotions and eating. Self-reported and experimentally induced changes in food intake under stress. Appetite.

[30] El Ansari W., Oskrochi R., Haghgoo G. (2014). Are students’ symptoms and health complaints associated with perceived stress at university? Perspectives from the United Kingdom and Egypt. Int. J. Environ. Res. Public Health.

[31] Papier K., Ahmed F., Lee P., Wiseman J. (2015). Stress and dietary behaviour among first-year university students in Australia: Sex differences. Nutrition.

